# Thermo-Physical Behaviour of Thermoplastic Composite Pipe for Oil and Gas Applications

**DOI:** 10.3390/polym17081107

**Published:** 2025-04-19

**Authors:** Obinna Okolie, Nadimul Haque Faisal, Harvey Jamieson, Arindam Mukherji, James Njuguna

**Affiliations:** 1 School of Computing, Engineering and Technology, Robert Gordon University, Garthdee Road, Aberdeen AB10 7GJ, UK; o.okolie@rgu.ac.uk (O.O.); n.h.faisal@rgu.ac.uk (N.H.F.); 2 Subsea 7, East Campus, Prospect Road, Arnhall Business Park, Westhill, Aberdeenshire AB32 6FE, UK; harvey.jamieson@subsea7.com; 3 SP Advance Engineering Materials Pvt Ltd., SP Centre, 41/44, Minoo Desai Marg, Colaba, Mumbai 400 005, India; arindam.mukherji@shapoorji.com; 4 National Subsea Centre, 3 International Avenue, Dyce, Aberdeen AB21 0BH, UK

**Keywords:** non-isothermal crystallinity, thermal stability, melt behaviour, functional group, crystallisation kinetics, anisotropy

## Abstract

Thermoplastic composite pipes (TCP) consist of three distinct layers—liner, reinforcement, and coating—offering superior advantages over traditional industrial pipes, including flexibility, lightweight construction, and corrosion resistance. This study systematically characterises the thermal properties of TCP layers and their compositions using a multi-method approach. Thermal analysis was conducted through differential scanning calorimetry (DSC) for isothermal and non-isothermal crystallisation, thermogravimetric analysis (TGA) for thermal stability, and Fourier transform infrared spectroscopy (FTIR) for material identification. FTIR confirmed polyethylene as the primary component of TCP, with compositional variations across the layers. TGA results indicated that thermal degradation begins at approximately 200 °C, with complete decomposition at 500 °C. DSC analysis revealed a double melting peak, prompting further investigation into its mechanisms. On-isothermal crystallisation kinetics, analysed at cooling rates of 10 °C/min and 50 °C/min, revealed an anisotropic crystalline growth pattern. Although nucleation occurs uniformly, the subsequent three-dimensional crystalline growth is governed more by the degree of supercooling than by the crystallography of the glass fibres. This underscores the importance of precisely controlling the cooling rate during manufacturing to optimise the anisotropic properties of the reinforced layer. This study also demonstrates the value of FTIR, TGA, and DSC techniques in characterising the thermo-physical behaviour of TCP, offering critical insights into thermal expansion, shrinkage phenomena, and overall material stability. Given the limited body of research on this specific TCP formulation, the findings presented here lay a foundation for both quality enhancement and process optimisation. Moreover, the paper offers a distinctive perspective on the dynamic behaviour, thermal expansion, and long-term performance of TCP in demanding oil and gas environments.

## 1. Introduction

Composite technologies are greatly preferred in a lot of industrial sectors, such as automotive, aviation, energy, etc. [1]. In recent years, reinforced polymeric composites have been used extensively in many engineering applications such as chemical, oil, and electricity. They use pipes that are made of polymers reinforced with fibres. In the energy industry, composite pipes are increasingly being used as an alternative to conventional steel-based pipes. This application of composite materials was developed in response to significant corrosion problems with the metallic pipes. Other reasons are the low density of composite pipes, which is about a quarter the weight of steel. This makes them an attractive choice of material, especially in applications where heavy handling equipment is not viable for installations, e.g., in remoted or deep-water conditions, offshore where the ease and speed of assembling a lightweight pipe are fundamental [2,3,4,5,6,7]. Composite pipes also offer a lower thermal expansion factor, superior strength-to-weight ratio and low friction factor [7,8].

Due to the increasing demands of the energy industry, factors such as high-temperature resistance and the need for damage tolerance and flexibility often affect the capabilities of composite pipes [3]. Composite pipes can have a pressure expansion that is 25 times greater than carbon and stainless steel and can potentially affect many design properties [7]. However, polymeric composites can degrade when exposed to certain environments that they can be brought into contact with during their service life. Temperature, pressure, and moisture are the most common service environment factors that fibre-reinforced composite pipe is exposed to [9]. Therefore, with composite pipes expected to solve the challenges with metal pipes in engineering applications, huge attention has been directed towards the manufacturing and testing of composite pipes.

In terms of material behaviour and characterisation for identifying manufacturing parameters, Alkan et al. [10] experimentally investigated the properties of HDPE/glass fibre composites with varying weight fractions, emphasising temperature as a major factor in material degradation. FTIR analysis identified chemical bonds and crystallinity, with HDPE exhibiting methylene C-H stretching vibrations near 2950 cm^−1^, C-H scissoring at 1460 cm^−1^, and a CH_2_ rocking mode at 720 cm^−1^. The band at 1303 cm^−1^ was linked to the amorphous nature of the polymer, while the weakness of the 1894 cm^−1^ band suggested low crystallinity. Increasing fibre content reduced transmittance. Behboudi et al. [11] studied PE and glass fibre composite membranes prepared via thermally induced phase separation. FTIR analysis showed an absorption peak at 720–740 cm^−1^ for the –(CH_2_)n– group in HDPE, while composite membranes exhibited a new peak at 1410 cm^−1^ (Si–CH=CH_2_, vinyl group). A broad peak between 3600 and 3100 cm^−1^ indicated OH presence due to glass fibres. Siddique [12] conducted ATR-FTIR analysis on neat LDPE, LDPE/MMT nanocomposites, and LDPE/OBM slurry nanocomposites, highlighting the role of structural OH and Si-O groups in identifying clay minerals. Maheswari et al. [13] explored the impact of fibre incorporation into HDPE, while Hu et al. [14] used FTIR to compare the valence bond structures of pure HDPE and cross-linked HDPE (XLPE), noting similar absorption bands between the two.

The use of DSC provides the released heat flow measurement (exothermic) and the evolution of the specific heat capacity. The derived heat flow will be utilised in studying the influence of temperature and time on the physicochemical state of the matrix. This dynamic and isothermal procedure characterises the glass transition temperature and kinetic behaviour of the material. The thermal characterisation of pure HDPE and HDPE composite through TGA and DSC was carried out by Awad et al. [15]. The experiment revealed that the addition of marble and granite dust particles increases the degradation temperature by 11 °C and reduces the loss rate of HDPE. Also, no significant change in melting point was observed. To investigate the effect of the degree of crystallinity in semi-crystalline thermoplastics on the properties of composite pipes, Batista et al. [16] used DSC to study different induced degrees of crystallinity in carbon fibre (CF)-reinforced polyphenylene sulphide (PPS) composites by using three different cooling rates during processing. The melting peak area of the DSC curves increases with the decrease in cooling rate due to the higher crystalline content in the PPS matrix. It is observed that the melting temperature increases from fast in air cooling, which indicates that the solid polymer matrix resulting from the latter cooling rate has better-ordered crystallites resulting from the longer cooling time. Therefore, a longer cooling time favours the alignment and folding of the polymer chains, forming regions more orderly.

Also, Wang et al. [17] investigated the melting and crystallinity behaviour of varying glass fibre-reinforced polypropylene composite. From the result, the crystallisation temperature gets high with increasing glass fibre content, but there is little variation in crystallisation temperature that makes the variation insignificant. Although the crystallinity shows a trend of increasing initially, it later decreases, since a small amount of GF could act as a nucleating agent in the system, promoting the heterogeneous nucleation of PP and causing the improvement of crystallinity. However, when the amount of GF was larger, the presence of GF prevented the α-spherulites of PP from expanding in all directions, thus resulting in a decrease in crystallinity. In contrast, a small amount of GF can provide the nucleation centre to induce the crystallisation at high temperatures, reduce the supercooling degree of the sample, speed up the crystallisation rate, shorten the production cycle and greatly save the processing cost. Particularly for thermoplastics that are semi-crystalline, the degree of crystallinity and crystalline morphology are the key variables in ascertaining the physical and mechanical properties of the end part. The matrix crystallisation is often not solely affected by the processing conditions; the presence of reinforcements also contributes [18]. Usually, the crystallisation process undergoes non-isothermal conditions during processing (industrial processes such as injection moulding and prepreg processing) [19]. To quantify the effects of the fibre presence on the crystallisation behaviour of the TCP, kinetic model parameters are utilised [18].

Despite the growth of TCP applications based on the benefits, detailed research on their specific physical and thermal properties remains limited. This gap in knowledge impedes the optimisation of processing parameters for manufacturing and limits the potential for quality improvement in the varying forms of the TCP. To address this, this article proposes a comprehensive set of characterisation methods aimed at obtaining reasonably accurate material properties for TCP, which are currently largely unknown. The proposed methodology employs a multi-faceted approach, combining material identification, thermal behaviour analysis, and crystallinity assessment. Specifically, FTIR, TGA, and DSC are utilised to investigate key aspects such as material composition, thermal stability and crystallinity kinetics for each layer. The primary objective and novelty of this article is to deepen the understanding and provide a comprehensive analysis of the thermal properties and behaviour of TCP, especially the glass fibre-reinforced PE for oil and gas applications. Therefore, this lays the groundwork for informing the development of optimised TCP manufacturing processing techniques. By deducing the thermal characteristics of TCP, this research contributes to providing valuable insights that can drive advancements in the design and manufacturing of high-performance composite pipes.

## 2. Experiment

### 2.1. Materials

**Figure 1:**
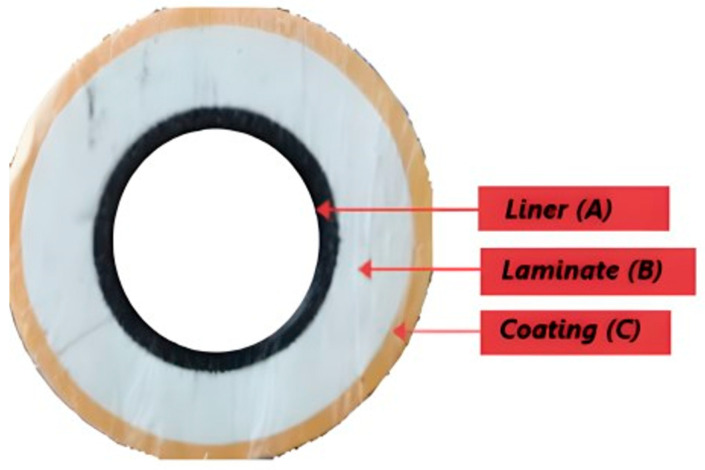
TCP pipe section with the lateral view displaying all the pipe layers.

A melt-fused TCP was donated for the purpose of this research. The pipe has a 50 mm ID and a 102 mm OD with a length of 1500 mm and is stored at room temperature. Also, from the database, this is a glass fibre-reinforced high-density polyethylene (HDPE) TCP with a burst pressure of 5 ksi and an operating temperature of 65 °C. It is made of 3 fully bonded layers: the inner, the reinforced, and the outer. The thickness of the inner layer is 4 mm, while that of the reinforced and outer layers is 17 mm and 4 mm, respectively, with a total thickness of 25 mm, as displayed in Figure 1.

The test samples were machined from the TCP using a diamond blade saw cut and fair-faced for a smooth surface finish. A set of 3 to 5 samples is prepared and stored at room temperature, with no pre- or post-treatment needed.

### 2.2. Sample Preparation

The test samples were machined from the thermoplastic pipe using a diamond blade saw cut and fair-faced for a smooth surface finish. Pellet size samples for characterisation were obtained using a Stanley knife from sections of the pipe. For TGA and DSC, 3 samples of the whole layer with similar properties were performed before doing each layer for general understanding and consistency of result. All the samples are stored at room temperature, and pre-treatment on the sample is not required.

### 2.3. Characterisation

Fourier transform infrared (FTIR) spectroscopy was conducted to identify the compounds present in a material based on their functional groups. The FTIR was performed on a NICOLET iS10 using the OMNIC software version 9.12 attached to attenuated total reference (ATR). Small solid samples stored at room temperature were used for each layer. The equipment was set for 32 scans within 400–4000 cm^−1^.

Thermogravimetric analysis (TGA): To assist in determining the processing conditions and how they can be used. TGA and differential scanning calorimetry (DSC) are used to understand the temperature and kinetic degradation during service. While the mass of each sample is not established, a sufficient sample size is required to fill the pan regardless of the uniform distribution for each layer. The onset of thermal degradation was measured at roughly 5 wt.% mass loss that correlates with temperature change. The pan used for the analysis was platinum-based, which is capable of withstanding a high temperature of roughly 800 °C and is mainly used for oxidation reactions. There is a variation in the mass of samples, but it lies within the range of 6–8 mg. The rate is at 10 °C/min with an end temperature set at 800 °C. Hence, the test was initiated at room temperature and increased to 800 °C, which is related to the weight loss percentage, as was noticed.

Differential scanning calorimetry (DSC): The DSC used in this study was the TA Q 100 series, and the sample was weighed and placed in an aluminium pan with a high melting point (660 °C), which was closed with a lid and crimped to facilitate improved thermal contact. The concept for this technique is the heat exchange difference between the prepared sample and the referenced empty sample. To increase heat transfer during the experiment, liquid nitrogen is used as the purge gas. The weight of the samples was measured with an electronic microbalance, and all samples were within the range of 6–10 mg as recommended for the equipment. The DSC procedure was carried out in 3 cycles with 6 steps: Cycle 1: Equilibrate at 0 °C, ramp up to 150 °C at 10 °C/min; Cycle 2: Equilibrate at 150 °C, ramp down to 0 °C at 10 °C/min; and Cycle 3: Equilibrate at 0 °C, ramp up to 150 °C at 10 °C/min. The temperature connected to the endothermic peak is termed the melting point (Tm), and that of the exothermic peak is the crystallisation peak (Tc). The combination of the conversion of the matrix heat of fusion at 100% crystallinity and melting peak regions generates a precise percentage of crystallinity (Xc).

(1) $$X_{c}=\left(\frac{\Delta H_{f}+\Delta H_{c}}{\Delta H_{100}}\right)\times 100$$

Here, $\Delta H_{f}$ is the fusion enthalpy (determined by experiment), $\Delta H_{100}$ is the heat of fusion at 100% HDPE polymer crystallinity, which was found to be 293 J/g from the literature [9], and $\Delta H_{c}$ is the enthalpy of crystallisation.

The DSC temperature was elevated from 10 °C to 160 °C, which is greater than the melt temperature at a heating rate of 10 °C/min, and left for 15 min to enable thermal history elimination in the sample. Subsequently, the samples are cooled to 10 °C at 2 different cooling rates, 10 °C/min and 50 °C/min, respectively. The non-isothermal crystallisation of the individual matrix is conducted with the DSC as the samples are heated from 10 °C to 160 °C at a rate of 10 °C/min and a 50 °C/min cooling rate.

## 3. Results and Discussions

### 3.1. Elemental Composition Analysis

**Figure 2:**
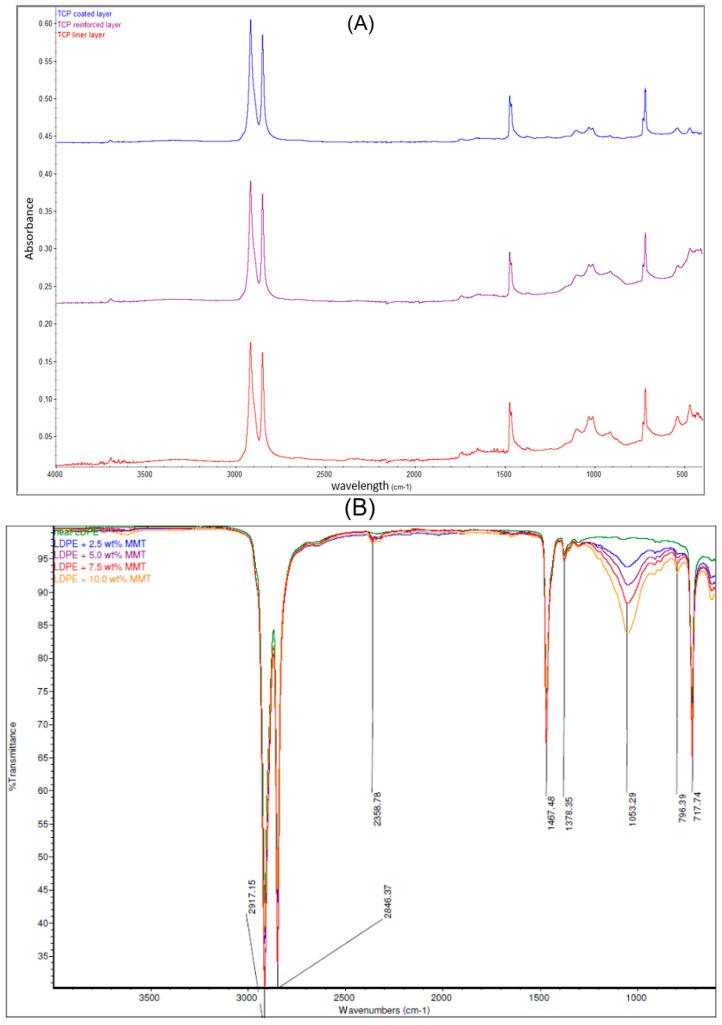

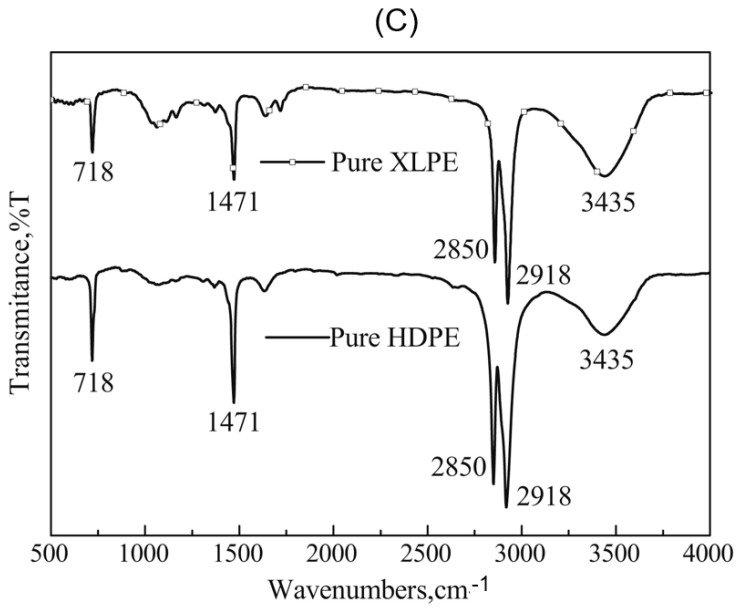
(**A**) ATR-FTIR spectra which indicate the composition of the liner, laminate, and coated layers. (**B**) Comparison of ATR-FTIR common scale spectra of LDPE and LDPE/MMT nanocomposites [12] and (**C**) the FTIR curves of pure HDPE and pure XLPE [14].

Each layer of the pipe was examined by FTIR spectroscopy attached to ATR to study the elements at the surface of the samples. The peak from the results provides detailed information on it, and the use of the library manager will also give a clue. In Figure 2A, the spectra of the inner layer sample are presented and show that it is a polyethylene functional group. From the studies of Maheswari et al. [13] and Mahmud [20], the slight absorption peak within 3500–3000 cm^−1^ indicates that this layer has little fibre and was treated to handle the conveyed fluid conditions. The peaks are four bands that are at the peaks of 2913.50 cm^−1^, 2846.66 cm^−1^, 1469.45 cm^−1,^ and 716.64 cm^−1^. The absorbance peaks at 2913.50 cm^−1^ and 2846.66 cm^−1^ represent the presence of a stretch vibration C-H bond with strong absorption for heavy presence, at the 90% absorbance between the 2000 cm^−1^ and 2500 cm^−1^ indicating an attachment to a primary amine, while the 1469.45 cm^−1^ and 716.64 cm^−1^ signify a bending vibrating C-H and C-H cis functional group in medium absorptions, respectively. All this indicates an alkene group attached to a primary amine. Using the work performed by Siddique [12] on LDPE, this layer is of the ethene family but not LDPE (see Figure 2B). However, from the FTIR curves obtained by Hu et al. [14] in Figure 2C that studied the thermal properties of both high-density polyethylene (HDPE) and cross-linked high-density polyethylene (XLPE), this layer fits more to HDPE.

Also, from the FTIR spectra in Figure 2A, the spectra of the reinforced layer suggest that the polyethylene, like the previous layer with almost similar peaks at 2913.50 cm^−1^ and 2847.09 cm^−1^, indicates a stretch vibration of the C-H group with strong absorption. Two primary amine groups are spotted at the peaks of 2359 cm^−1^ with a stretch vibrating medium absorption and 1649.74 cm^−1^ with a bending vibrating strong absorption. The 906.75 cm^−1^ and 717.18 cm^−1^ peaks indicate C-H trans and cis isomers, respectively. The curve within 3000–3500 cm^−1^ indicates the presence of fibre and indicates that this layer was treated to enhance the fibre and matrix compatibility [10,11]. It can be finalised that this is an alkene functional group attached to two amine groups with strength in absorption, and using the study by Siddique [12], there is spectra similarity with LDPE, which indicates a lower density of polyethylene compared to the other layers. However, from Figure 2A, although the peaks at 2912.86 cm^−1^, 2846.39 cm^−1^, 1470.03 cm^−1,^ and 717.02 cm^−1^ are similar to the inner layer spectra, the amine functional group is absent. No absorption is noticed within 3000 to 4000 cm^−1^ which indicates that the fibre content in this layer is the lowest across all layers [10,11]. It also fully matches the FTIR spectra of HDPE as the polymeric matrix [10], and the layer is treated to be less susceptible to external material attack or degradation. This further validates the thought that the vital element of the TCP sample is polyethene. Also, the presence of the amine group can be attributed to siliconization of glass fibre, where a thin layer of silane group (3-aminopropyltrimethoxysilane) is added to glass fibre to make it greatly hydrophobic and improve fibre-to-matrix compatibility. Further tests using X-ray photoelectron spectroscopy (XPS) will show the elements present in the samples and their possible bonds.

### 3.2. Thermal Degradation

**Figure 3:**
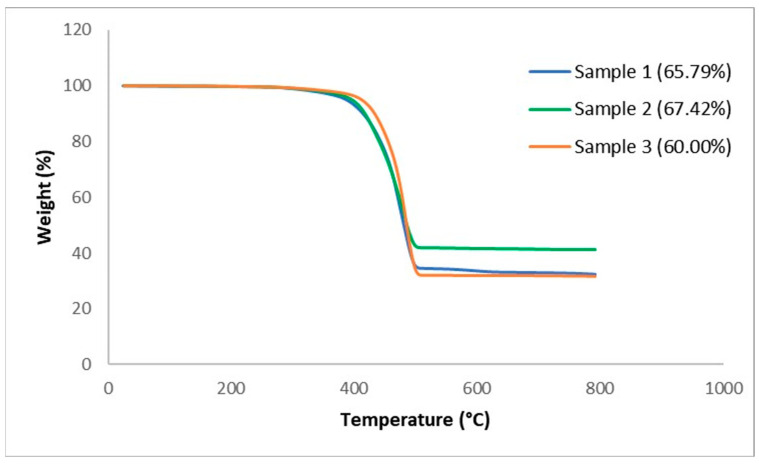
TGA thermograph for full samples.

The thermal stability of the TCP and each was studied using TGA. Figure 3 shows the TGA thermograms of weight change of the full TCP with two samples at a similar condition with the rate of 10 °C/min.

The thermograms reveal an almost similar process for both samples with only one major weight loss transition. Although the onset temperature of both samples is roughly 200 °C, most of the degradation seems to have occurred at the temperature range of 200–600 °C. From the final residue weight, it is noticed that the degraded portion differs, as that of sample 1 is 65.94%, sample 2 is 67.42%, and sample 3 is 60.00%. This is attributed to differences in mass for each layer in the full samples, with all indications that the glass fibre remains stable after roughly 625 °C. With these results, an attempt can be made to estimate the fibre volume fraction based on the resin burn-off technique from the fibre weight to composite weight ratio presented in Table 1. From Table 1, it can be stated that the fibre weight percent of the composite is within the range of 33–40%. To clarify this, Figure 3 shows the weight change for each layer of the TCP.

Through the glass fibre residue left after the matrix degradation, the matrix weight can be deduced from the sample mass. All the samples undergo a similar degradation process within the temperature range of 200–600 °C with a similar single major weight loss transition. Aside from the sample mass, other factors that influence the matrix degradation observed from this procedure are differences in thermal stability and degradation kinetics of the matrix between the layers. Also, the distribution of the matrix and fibre across the layers is also influential. It is expected that layers within the sample with a more uniform distribution of components exhibit a more consistent degradation profile.

**Figure 4:**
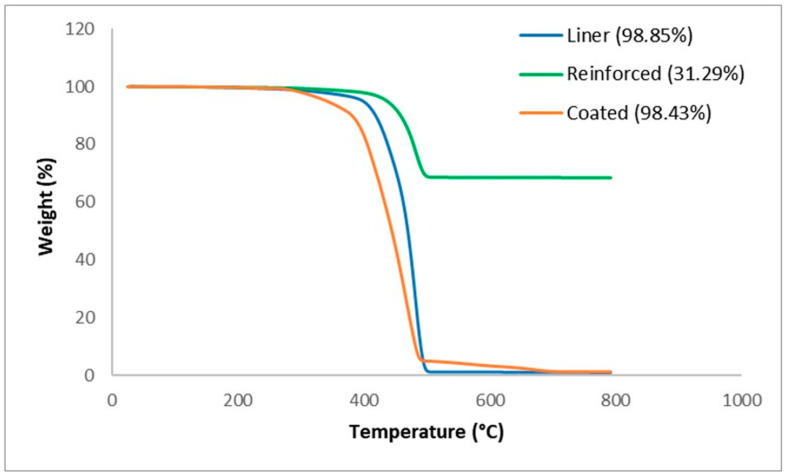
TGA thermograph of the liner, reinforced, and coated layers.

From Figure 4, it is deduced that each layer also has one major process path. At 500 °C the coated layer has a bulk degradation, which implies that the layer is thermally unstable at this temperature. However, there is still a small fraction that is thermally stable at higher temperatures, which can be stated to be either fillers (fibres) or impurities.

The thermograph for the liner layer signifies a more thermally stable behaviour, which is understandable as it is expected to handle high-temperature fluids. The bulk degradation of the liner layer ends at roughly 700 °C. From observation, it is evident that another component is present that makes it different from the coated layer. The reinforced layer further confirms the assertion that there is a massive glass fibre fraction present in the reinforced layer, which is roughly at 0.68, and this directly influences the material properties of the composite. Also from this experiment, it is revealed that the addition or increase in fibre content increases the degradation temperature, which is confirmed in similar work performed by Awad et al. [15]. Basically, the coated layer undergoes bulk degradation at 500 °C, which infers that the polymeric matrix in this layer has a lower thermal stability at higher temperatures and lacks sufficient thermal stabilising agents within. This behaviour indicates that the coating is likely made up of substances that cannot withstand high thermal stress, resulting in substantial degradation. Although the fibre reinforcement residue is obtained, it contributes a minor fraction to resist bulk degradation at higher temperatures. Similar to the coated layer, the thermal stability behaviour of the liner layer is relatively close but with the presence of slightly more fibre reinforcement and thermally stable additives. However, the reinforced layer exhibits a substantially higher thermal stability among the three layers; this can be attributed to the high glass fibre content. Glass fibres are renowned for their excellent thermal and mechanical properties. The increased glass fibre reinforcement in the composite structure increases the resistance to degradation at high temperatures. The fibre fraction, quantified at roughly 0.68, directly influences the thermal performance. The fibres impede the propagation of thermal degradation by acting as barriers within the matrix, thereby enhancing the overall stability of the reinforced layer. Furthermore, this is experimentally alluded to by the studies of Awad et al. [15], which show that increasing the glass fibre content raises the degradation temperature of global composite, a phenomenon attributed to the ability of the glass fibre to absorb and dissipate thermal energy effectively.

### 3.3. Crystallisation and Melting Behaviour

**Figure 5:**
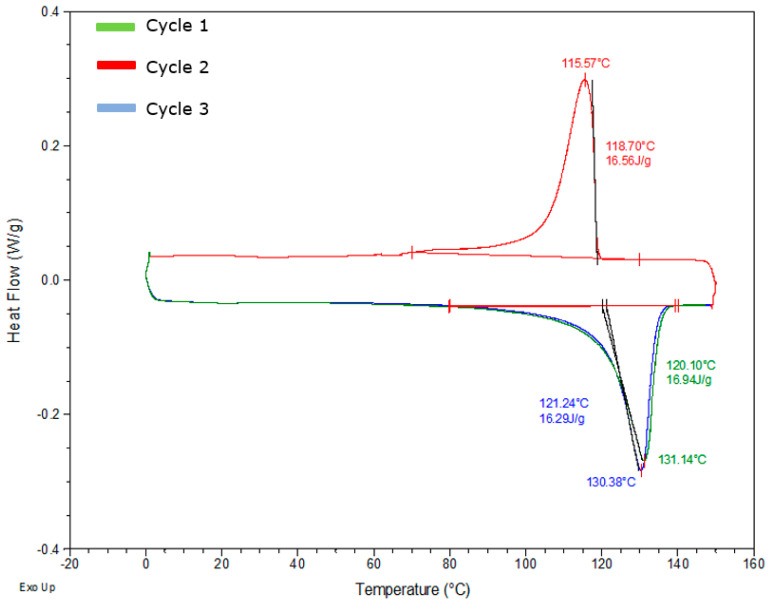
DSC thermogram of the full sample with all layers at the glass transition temperature, melting temperature, and crystallisation temperature.

To determine the crystallisation and melting process of the TCP sample, DSC was performed in three cycles for all the samples, as earlier stated. A controlled amount (6–10 mg) of the combined three layers is first carried out to understand the general thermal behaviour fully. From Figure 5, all the samples undergo glass transition within 5 °C at cycle 1, and the melting range of the samples is displayed as an exothermic peak. Recrystallisation occurs in cycle 2, where the crystallisation temperature is deduced at the endothermic peak. Unlike cycles 1 and 3, which undergo melting, cycle 2 is the crystallinity curve for the sample where cooling occurs. In cycle 3 the glass transition does not change. However, there is a second melting peak which indicates that the HDPE crystals are orientated on a fibre surface; hence, the introduction of fibre causes this phenomenon. A possible reason for this based on Miao et al. [21] is the presence of two forms of crystals with varying thickness. This is because, in similar heating conditions, crystals of the same polymer created after recrystallisation should have similar melting points as the glass transition. This should mean that the high melting temperature peak of HDPE crystals created on the fibre surface at varying crystallisation temperatures can be seen after melting recrystallisation. To fully investigate these hypotheses, partial melting testing will be needed to understand the fine structure of HDPE crystals with the fibres.

Furthermore, the enthalpies and temperatures of the cycles in the other DSC samples follow the same path and are within a similar range. To derive the degree of crystallinity of the samples, the melting temperature at cycle 1 is considered here, which is presented in Table 2 using the crystallinity equation as provided.

From Table 2, a relationship between Xc and the sample mass as a reduced mass tends to increase the Xc value. This can be attributed to increasing nucleation and crystal density increasing with increased mass. This increased mass limits the macromolecular chain movements, and this restricts the crystal growth during the procedure. There is also an expectation of inaccuracy in Xc, as the amount of fillers that include fibres was not considered. The layers of the TCP were also studied in a similar procedure as stated earlier, to understand the thermal behaviour at the nip point.

**Figure 6:**
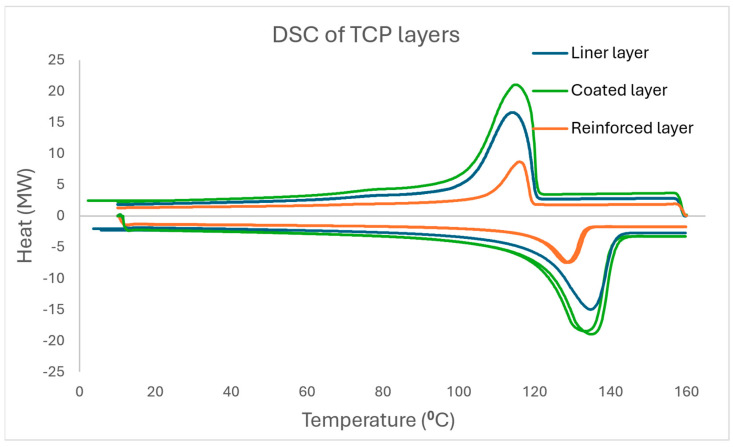
DSC thermogram displaying the glass transition temperature, melting temperature, and crystallisation temperature of the liner, reinforced, and coated layers at 10 °C/min.

From the DSC thermograms in Figure 6, the coated layer has a slightly higher glass transition temperature range compared to the other layers, while the reinforced layer has a slightly lower glass transition temperature range, and this indicates the level of amorphous portion present in the samples and the possibility of a component acting as a plasticiser in the reinforced layer. All layers still exhibit a double melting peak at close temperatures, which signifies that the polymer matrix is of the same family throughout the TCP. Also noticed is that the reinforced layer has the lowest enthalpy and melting point compared to other layers, which are within close range, and this is because this layer has the highest fibre content among the layers and also may support the observation that the polyethylene matrix here has a lower density than HDPE. The degree of crystallisation of the layers is highlighted in Table 3 below.

The massive fibre presence in the reinforced layer is reflected, as it has a major effect on the Xc value [22]. The fibre has a nucleating effect which facilitates crystallisation. To buttress an earlier statement, when crystal growth is restricted in the fibre plane based on the nuclei being tightly packed, the growth will then be in the surface direction of the nucleus, and this is termed transcrystallisation. Generally, when crystallisation occurs with both polymer and shear flow across the fibre surface, transcrystallisation is bound to occur. Therefore, with all the layers, especially the reinforced layer, the impregnation of the fibre and consolidation of the layers can be suggested to force transcrystallisation. This is confirmed based on the reinforced layer having the highest degree of crystallinity and crystallisation temperature compared to the other layers, which are part of the reasons for the low enthalpies of the layer. Basically, the coated and liner layers appear to have similar thermal behaviour, and this is attributed to a lower filler/fibre content present in them compared to the reinforced layer.

### 3.4. Non-Isothermal Crystallisation Behaviour

**Figure 7:**
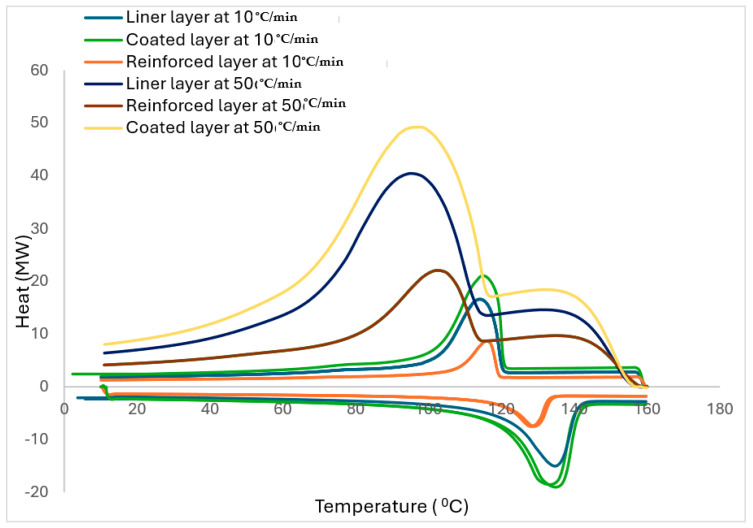
The DSC curves of the layers for the two cooling rates of 10 °C/min and 50 °C/min.

As previously stated, the fillers in polymers significantly influence the crystallisation behaviour of the reinforced polymer composite. The crystallisation process is carried out either in isothermal or non-isothermal conditions. However, due to the ease of theoretical analysis of results, the investigations of isothermal conditions are common. Practically, polymers and their composites are subjected to non-isothermal processes; hence, the non-isothermal crystallisation conditions are more useful. The DSC curves of the layers for the two cooling rates of 10 °C/min and 50 °C/min are displayed in Figure 7. The crystallinity curve for 50 °C/min is added from Figure 6.

Certain kinetic parameters can be obtained from non-isothermal crystallisation exotherms, such as the onset temperature (T_onset_), which is the temperature of the region where the tangents of the bassline and the high-temperature area of the exotherm cross. Also, the peak temperature (T_peak_), the crystallisation enthalpy (ΔH_c_) and the half-crystallisation time (t_1/2_) are the times needed for the crystallisation to be at 50% and can be obtained and listed in Table 4 at the varying cooling rates (φ).

As seen in Table 4, there was a reduction in T_onset_, T_peak_, enthalpy and *t*_1/2_ variables for all the layers with an increase in φ; hence, there was no critical difference in these variables with the variations in fibre volume, as there is a similar trend at isothermal conditions. The range of T_onset_ for all the composites was within 116.31–118.70 °C. However, the higher cooling rate (50 °C/min) had the lower temperature for maximum crystallisation. Similarly, the T_peak_ for the reinforced layer for both cooling rates is higher than the other layers, which confirms that increased fibre volume also increased the crystallisation rate of the TCP while both the liner and coated layers seem to have approximately the same amount of fibre volume. In addition, it was deduced that the increase in φ generated a broader difference range between T_onset_ and T_peak_. This confirms that at a faster φ there is insufficient time for nuclei activation, while for slower φ, it is the reverse at higher temperatures. Hence, it is at lower temperatures that activation occurs for faster φ, which is confirmed through the t_1/2_. As displayed in Table 4, φ increased as ΔH_c_ reduced, and the increased fibre volume presence in the reinforced layer significantly shifted the ΔHc lower, which aligns with the earlier assertion that the fibre serves as a nucleating agent for the HDPE matrix. However, it can be beneficial to determine the optimal fibre-to-matrix ratio that can influence the ΔH_c_ values. Furthermore, the HDPE chain growth activity was limited by the fibre, and this subsequently influenced the matrix crystallisation process in the reinforced layer in comparison to the other layers with minute fibre presence.

As aforementioned, variables from non-isothermal crystallisation kinetics are necessary benchmarks in preparatory processing. Also, there is a lack of available research on melting behaviour for non-isothermal crystallisation of fibre-reinforced polymers, which makes it worthwhile to investigate. Consequently, the DSC curves for the layers with the two cooling rates are measured and displayed in Figure 7. As anticipated, the conventional crystalline peaks are deduced from the DSC curves. In addition, the crystalline temperatures marginally became lower with the increased φ (see Table 4 for the related crystallinity variable summary). Thereafter, the relative crystallinity (X_t_) values for the layers are computed initially using the following equation.

(2) $$X_{t}=\frac{H_{T}}{\Delta H_{C}}=\frac{\int_{T_{0}}^{T}\left(\frac{\mathrm{dH}}{\mathrm{dT}}\right)\mathrm{dT}}{\int_{T_{0}}^{\infty }\left(\frac{\mathrm{dH}}{\mathrm{dT}}\right)\mathrm{dT}}\times 100\%$$

Here, $\Delta H_{C}$ and $H_{T}$ refer to the generated heat during the entire crystallisation process and at a temperature of T, respectively, while T_0_ is the first crystallisation temperature and dH/dT represents the heat flow rate. The derived results are plotted as curves which are depicted as the crystalline degree transition of the layers with temperature variations. Here, $\Delta H_{C}$ and $H_{T}$ refers to the generated heat during the entire crystallisation process and at the temperature of T, respectively, while T_0_ is the first crystallisation temperature and dH/dT represents the heat flow rate. The derived results are plotted as curves which are depicted as the crystalline degree transition of the layers with temperature variations.

**Figure 8:**
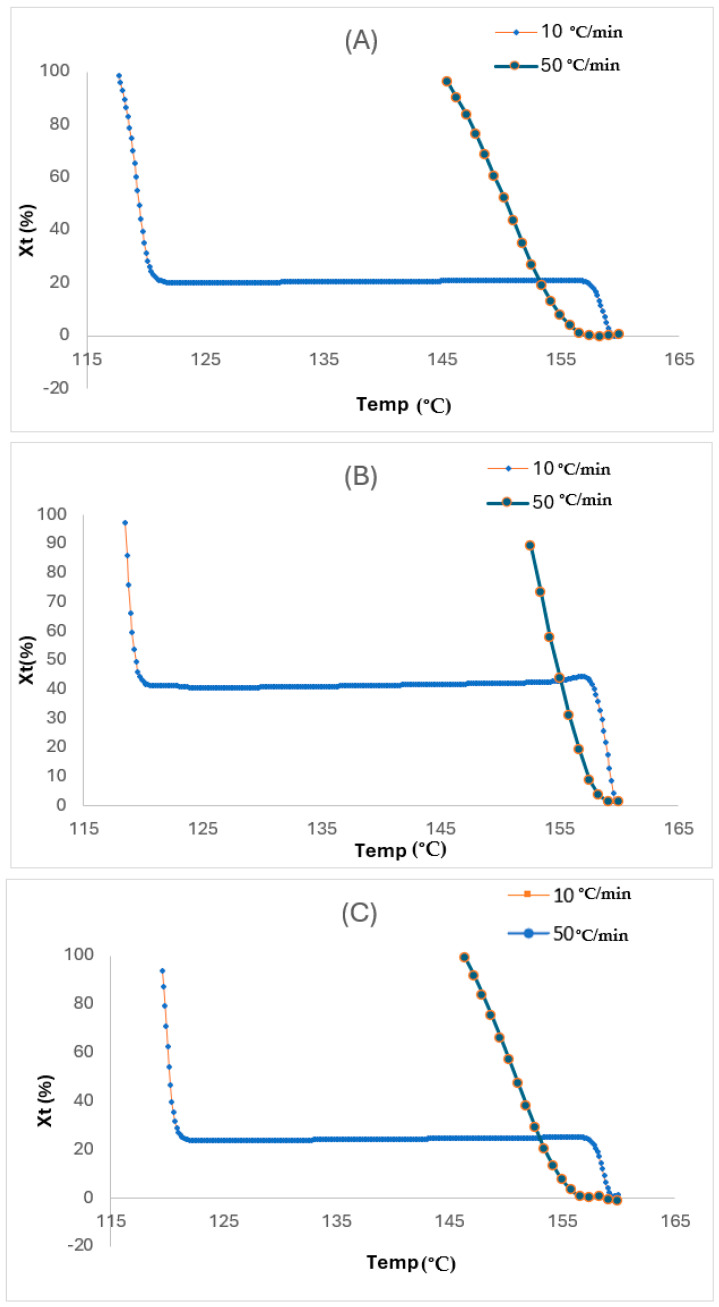
Plots of relative crystalline fraction at varying temperatures for (**A**) liner, (**B**) reinforced, and (**C**) coated layers.

For X_t_, it was derived from the region of the exothermic peak through the analysis of the non-isothermal crystallisation from DSC. From the relative crystalline fraction plots in Figure 8A–C, the X_t_, which is a function of temperature, is depicted. Although the crystallisation time for all the layers may be slower at the start for the faster rate, it eventually turned out faster as the X_t_ values became lower than those for the slower rate at the same crystallisation temperature conditions due to the shorter crystallisation temperatures at the faster rate. Therefore, the crystallisation temperature for the TCP elevates with an increase in the glass fibre content. This was linked to the ability of the fibre to act as a nucleating agent in the HDPE matrix. Also, this temperature reduces when the cooling rate is increased, and this is due to the high mobility of the polymer molecules from the cooling rate increase, which impedes the crystallisation ability. As observed, both the cooling rate and degree of crystallinity from the melt phase have significant impacts on the quality of the TCP produced through melt fusion bonding. Whereby the critical crystallinity is attained prior to the cooling phase during manufacturing, defects from the consolidation phase or device can be induced. Therefore, it is imperative that the non-isothermal crystallisation kinetics governing the TCP is understood and established. Despite the conventional DSC method used to ascertain the isothermal crystallisation of thermoplastics, the Avrami model has been used in quantifying both the isothermal and non-isothermal crystallisation behaviour [23]. Contrastingly to the isothermal analysis, the Avrami exponent (n) and the non-isothermal crystallisation rate constant ($Z_{t}$) are obtained through the $\ln\left[-\ln\left(1-X_{t}\right)\right]$ vs. ln t plot. This model is based on the assumption that crystallisation develops at a constant temperature.

#### 3.4.1. Avrami Method

The Avrami equation can also be used in analysing non-isothermal crystallisation by considering the characterisation of the material of interest. To describe the crystallisation rate for thermoplastics in isothermal conditions, the generic Avrami model is used based on DSC with a simplified assumption of constant temperature. The Avrami equations can be expressed as follows.

(3) $$1-X_{t}=\exp\left(-Z_{t}t^{n}\right)$$

$X_{t}$ here can be defined as follows:

(4) $$X_{t}=\frac{\int_{T_{0}}^{T}\left({\mathrm{dH}}_{c}/\mathrm{dT}\right)\mathrm{dT}}{\int_{T_{0}}^{T_{\infty }}\left({\mathrm{dH}}_{c}/\mathrm{dT}\right)\mathrm{dT}}\in \left(0,1\right)$$

Herein, $dH_{c}/dT$ represents heat flow at temperature T and $T_{0}$ and $T_{\infty }$ are for the onset and end of the crystallisation temperature, respectively. A double logarithmic format is recommended for a simplified handling of data as follows.

(5) $$\ln\left[-\ln\left(1-X_{t}\right)\right]={\mathrm{lnZ}}_{t}+\mathrm{nlnt}$$

$Z_{t}$ refers to the crystallisation rate constant, n is the Avrami exponent in the non-isothermal process, and t represents time. For non-isothermal crystallisation, Jeziorny [23] recommended that the effect of the cooling rate (R) be considered and consequently corrected $Z_{t}$ as expressed.

(6) $${\mathrm{lnZ}}_{c}=({\mathrm{lnZ}}_{t})/R$$

**Figure 9:**
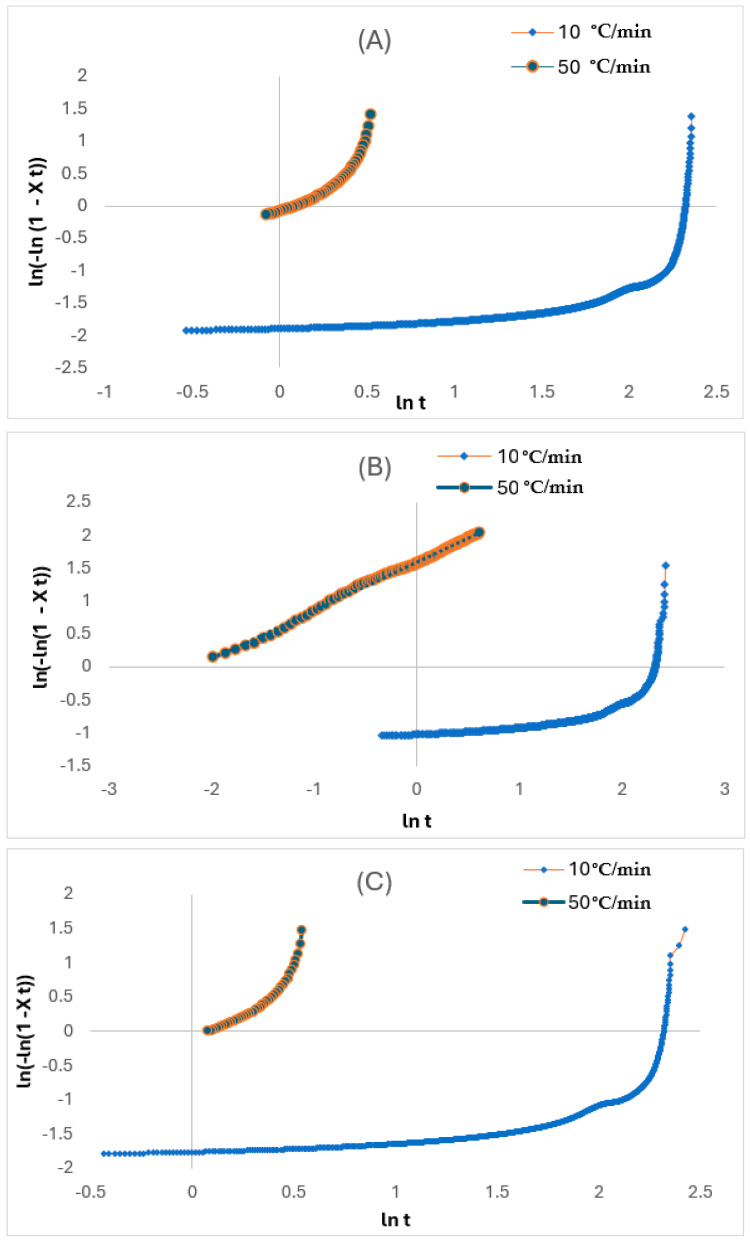
Plots of $\ln\left[-\ln\left(1-X_{t}\right)\right]$ against ln t for (**A**) liner, (**B**) reinforced, and (**C**) coated layers.

$Z_{c}$ represents the revised kinetic crystallisation rate constant. The time taken to achieve 50% of relative non-isothermal crystallinity is the halftime ($t_{1/2}$), which is given by the following:

(7) $$t_{1/2}={\left[\frac{\ln2}{Z_{t}}\right]}^{\frac{1}{n}}$$

based on the plot of $\ln\left[-\ln\left(1-X_{t}\right)\right]$ against ln *t* (Figure 9A–C) for non-isothermal crystallisation of the layers at each φ, where t, which is the crystallisation time, is derived from the following equation:

(8) $$t=\frac{T_{0}-T}{R}$$

Here, T and $T_{0}$ refer to the temperature at any t and the whole crystallisation process, respectively, while R is the cooling rate. The values for Z_t_ and n in Table 4 are determined from the intercept and slope of the lines, respectively. There were considerable variations of temperature in the non-isothermal crystallisation, and this influenced the nucleation and spherulite growth based on being temperature reliant. Hence, the n and Z_t_ parameters do not share a similar interpretation with isothermal crystallisation.

As seen in the plots from Figure 9A–C, the plots are derived up to a high degree of conversion from the X_c_ (roughly 90%). This implies that the non-isothermal crystallisation of the various layers can be interpreted through the Avrami model, and the Z_c_ and n parameters can be used to thoroughly characterise this process. The curve-shaped appearance, in accordance with Xu et al. [24], attributed this based on the high degree of conversion. Whereby the additional crystallisation phase of the polymer molecules occurs inside the already existing crystalline entities, also termed the secondary crystallisation process. This finding by Xu et al. [24] also deduced that faster cooling rates enable the faster cooling rate that creates a wider temperature range between *T*_onset_ and *T*_onset_. The decrease in enthalpy and t_1/2_ at higher cooling rates supports the concept that faster cooling restricts polymer chain alignment, causing anisotropic crystal growth. This is based on crystallisation accelerating faster, but the degree of crystallinity reduces because of insufficient time required for crystal growth and nuclei activation. However, the bulk matrix is not involved in the development of newly formed crystals (primary crystallisation process) as wholly noticed during the initial phase of the process. The n values vary from 0.5 to 2.5 with the increase of cooling rate for both the coated and liner layers. This can indicate that the non-isothermal crystallisation process for the layer is characterised by the combination of a 3D spherical growth mechanism and a thermal nucleation process for the crystalline units [25,26,27]. Therefore, it can be stated that the increase in fibre content results in a decrease in n value, as that of the reinforced layer, from 0.42 to 0.73, and Boukettaya et al. [27] have observed similar results. This indicates that there is a shift from being 3D to more limited anisotropic growth with increasing fibre content. Moreover, the dual role of the fibre in chain mobility limitation and nucleation growth creates a directional reliance in crystal growth which causes anisotropy. Huang [28] attributed this reduction to the geometric change of the crystalline units, as the existence and rise in fibre content within the composite material do not enable these units to easily form in all directions. The latter highlighted finding of Xu et al. [24] is consistent with the findings by Huang [28] that the reduction in the n value can be attributed to the geometric limitations of the fibre that impedes isotropic crystal growth.

Considering the entire materials, the n value reduces with elevation of the cooling rate, which is the expected result, as the increase in n value decreases the crystallisation time, which subsequently enables the crystalline units to be wholly created during the crystallisation phase. Also, results from Table 4 indicate that the kinetic crystallisation rate (Z_c_) visibly increases with an increase in cooling and fibre content as the reinforced layer. This result confirms what was previously explained, which suggests that the presence of the glass fibre in the bulk HDPE matrix bolsters the crystallisation phase [29]. However, the assumption for the Avrami model that the crystallisation process develops at constant temperatures infers that if there are poor corrections, the Avrami model is insufficient for defining the non-isothermal crystallisation kinetics. This is broadly acknowledged, and from this consideration, there has been a modification to this model, the most notable being the Ozawa [30] and Mo [31] methods.

#### 3.4.2. Ozawa Method

This is based on the rate-dependent process of non-isothermal crystallisation with the assumption that crystallisation happens at a constant cooling rate. The effect of the cooling rate was used by Ozawa [30] to expand the Avrami equation for describing kinetics. In comparison to the Avrami model, the key difference is that the cooling rate variable replaces that of time. An equation was derived through assuming that the non-isothermal crystallisation comprises minute isothermal crystallisation phases, and this equation is expressed as follows:

(9) $$\ln[-\ln\left(1-X_{t}\right)=\ln K\left(T\right)-m\ln R]$$

m refers to the Ozawa exponent, which relies on crystal growth and nucleation mechanisms, and $K\left(T\right)$ is the cooling function linked to the crystallisation rate constant. For this method, at a specific temperature, the kinetic (Z_t_) and linear (m) parameters can be determined through the intercept and slope, respectively. Therefore, lnR can be represented as Inφ.

**Figure 10:**
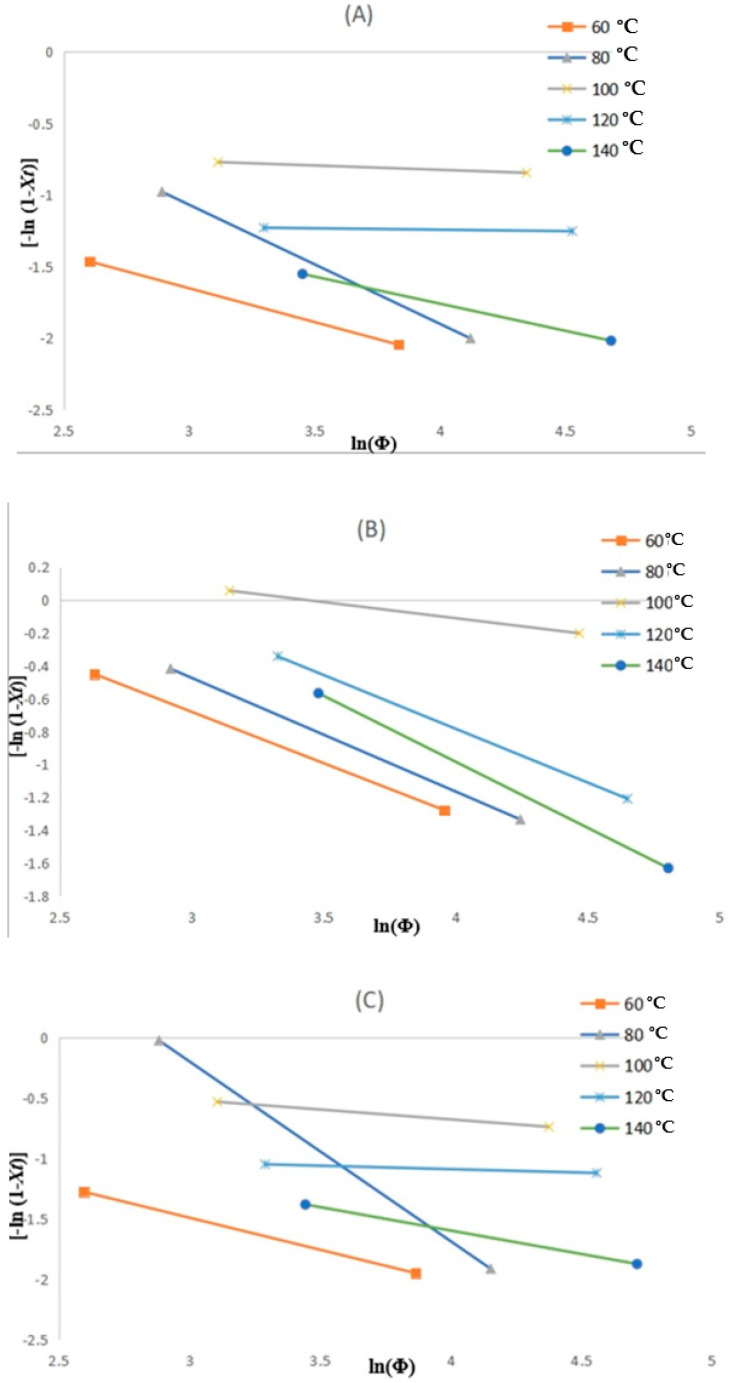
The Ozawa plots of ln [-ln (1-Xt)] vs. ln(Φ) for non-isothermal crystallisation of (**A**) liner, (**B**) reinforced, and (**C**) coating layers.

Figure 10A–C depicts the changes for the liner, reinforced and coated layers, respectively, where the resulting kinetic parameters are summarised in Table 5. The temperature range of 60 to 140 °C is derived from the crystallinity peak from the crystallinity curve in Figure 7. As noticed in the results considering the layers, variations in slope with temperature are deduced, and this implies that the m parameter is not a constant with temperature during crystallisation. This has been attributed to where the crystallisation process undergoes varying cooling rates at possibly different phases for a specific crystallisation temperature. Actually, at the elevated cooling rates, the crystallisation will be at the initial phase, and it reduces when the process is at the end. However, these uncertainties place the Ozawa model in dispute and are beyond the scope of this study.

#### 3.4.3. Mo Method

To formulate a more precise equation for describing the non-isothermal crystallisation procedure, the method proposed by Liu et al. [31] is a different kinetic equation for addressing non-isothermal crystallisation, as the Ozawa equation is combined with the Avrami equation to obtain a final equation as follows.

(10) $$\mathrm{lnR}=\mathrm{lnF}\left(T\right)-\alpha \mathrm{lnt}$$

where $F\left(T\right)={\left[K(T)/Z_{t}\right]}^{1/m}$.

**Figure 11:**
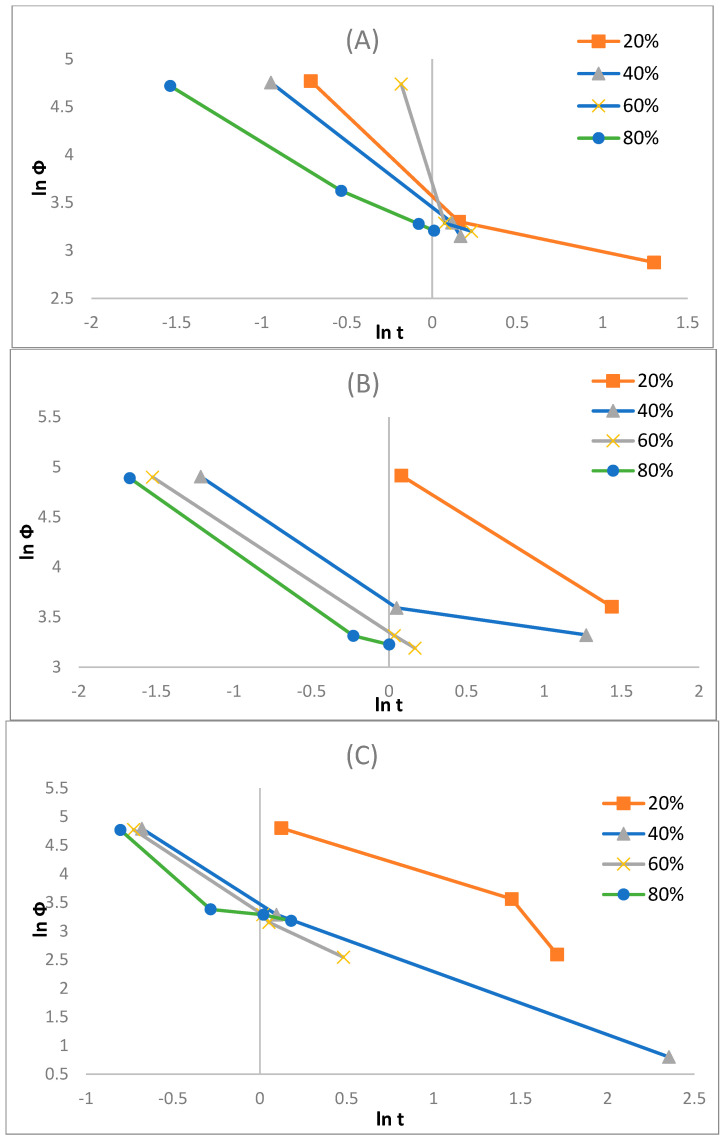
Mo’s plots of (**A**) liner, (**B**) reinforced, and (**C**) coating layers.

Here, α represents the slope, and $F\left(T\right)$ represents the cooling rate value selected at a specific crystallisation time when the process results in a certain degree of crystallinity, which is estimated by the intercept. With a reducing $F\left(T\right)$ value, the crystallisation rate increases. Hence, $F\left(T\right)$ has a distinct practical and physical connotation. The differences of ln (φ) vs. ln (t) at specific relative degrees of crystallinity Xt, which here are at 20%, 40%, 60%, and 80% (see Figure 8A–C), for the different composite layers considered in this research are displayed in Figure 11A–C, and Table 6 summarises the parameters.

The α values for each layer increased with elevated X_t_; however, the physical influence of α is unclear. In comparison to the F(T) values for the varying samples, it was deduced that the liner and coated layers are larger than the reinforced layers. This result can be interpreted to mean that the crystallisation rate of the reinforced layer was faster than that of the liner and coated layers, which is in accordance with the Avrami model. Boukettaya et al. [27] obtained a similar result, which infers that the F(T) reveals the influence of improving the crystallisation for the reinforcement stage in the HDPE matrix in the composite material. It seems that the introduction of glass fibre and its content increase in the bulk HDPE matrix have enabled the crystallisation phase, which proves their ability to nucleate, which is alluded to in the study by Yuan et al. [32]. Therefore, the fibre reinforcement in the reinforced layer enhances crystallisation rates, but due to the orientation limitations, anisotropy is introduced.

In addition, from Table 6 it appears that the crystallisation phase is at an advantage for the initial phase at lower X_t_ values. Typically, this is expected, as at the initial phase, the matrix is at a nearly complete melt state with a great molecular mobility, which consequently promotes the crystallisation phase. At higher X_t_ values with an increase in crystallisation time, this mobility decreases, and hence, the crystallisation process becomes more complex to derive, which in turn increases the F(T). Furthermore, for any sample, the ln F(T) values increase with an X_t_ increase, which implies that an elevated cooling rate can be utilised within a unit crystallisation time at a specific degree of crystallinity and insinuates that the greater the X_t_ is, the more complex the crystallisation will become.

In summary, the anisotropic behaviour of the TCP is significantly governed by the non-isothermal crystallisation process. Although the fibre contents serve as nucleating agents that enable anisotropy, the cooling rate is significantly dominant for dictating the orientation and degree of crystallisation. The crystallisation temperature for the TCP elevates with an increase in the glass fibre content. This was linked to the ability of the fibre to act as a nucleating agent in the HDPE matrix. Also, this temperature reduces when the cooling rate is increased, and this is due to the high mobility of the polymer molecules from the cooling rate increase, which impedes the crystallisation ability.

Therefore, there is an interplay between the fibre content, crystallisation behaviour and cooling rate that significantly influences the anisotropic properties of the TCP. Herein, the increased cooling rate creates a lower level of arrangement of the crystalline structure. This has the potential to increase the anisotropy due to the variations of mechanical properties across different orientations and the uneven stress distribution. This implies that the polymer chains align along the fibre orientation with a lower cooling rate, which increases the crystallinity more in the fibre-aligned orientation rather than the transverse direction, making it an anisotropic crystallinity. Furthermore, the faster cooling rate causes poor interfacial bonding because of lower crystallinity that increases anisotropy as the transverse properties weaken. Liu et al. [31] deduced this and re-emphasised the importance of the cooling rates for deducing the crystallinity. From the crystallisation kinetics studies, the slower cooling rate encouraged crystallinity with improved interfacial bond that can reduce anisotropy. To optimise the fibre alignment and the crystallinity behaviour of the TCP, appropriate processing conditions should be utilised to achieve the desired mechanical and thermal properties. This should be achieved by significantly reducing the cooling time without compromising the material quality post cooling in comparison to both the faster/maximum and slower/minimum cooling rate regimes. Therefore, there should be a trade-off between attaining optimal crystallinity and anisotropy.

For the prediction of non-isothermal crystallisation kinetics for each TCP layer, some prevalently used theoretical models have been considered. The observed findings imply that the Avrami analysis modified from Jeziorny and the Mo method proficiently described the TCP crystallisation kinetics. Contrastingly, the Ozawa model failed to proffer a thorough description of the non-isothermal crystallisation. The Avrami method enabled the identification of Z_t_ for the crystallisation rate of all the layers and confirmed that the glass fibre served as a nucleating agent, which is consistent with the findings from the Mo method, where the estimated parameters suggest that the non-isothermal crystallisation of all the layers correlates to a 3D growth for homogeneous nucleation. That means the non-isothermal crystallisation can be directly linked to the anisotropic. This is because although the nucleation from the non-isothermal crystallisation is homogeneous and is initiated uniformly, this growth can exhibit anisotropy. However, this growth cannot be directly attributed to glass fibre because it has low crystallographic properties but the supercooling rate of the process. Therefore, it can be stated that to optimise the anisotropic characteristics of the reinforced layer, the cooling rate is essential.

TCP manufactured through melt fusion bonding has been investigated by DSC, and the obtained results indicate that there were varying crystallisations with the presence of glass fibres in the composites and at varying cooling rates. Additionally, the T_peak_ values for the reinforced layers were slightly greater than that of the liner and coating layers, which indicates that the presence of glass fibre increased the crystallisation rate of the HDPE matrix, which implies that the fibre served as a nucleating agent for the matrix. However, the ΔH_c_ of the reinforced layer was significantly lower than the other layers, which implies that the fibre limited the activity of the HDPE chains and influenced the HDPE crystallisation during the process. In conclusion, the non-isothermal crystallisation kinetic features of each layer change with the glass fibre content and fibre orientation. While all three methods attempt to model crystallisation behaviour, the Avrami and Mo methods successfully describe the non-isothermal crystallisation kinetics of the TCP. Hence, the models reveal the competing effects of fibre nucleation and cooling rate in inducing anisotropy. In addition, these models capture the impact of the composite structure and processing conditions on crystallisation kinetics and, consequently, on material properties such as the interfacial strength. Further research should be on advancing theoretical models for non-isothermal crystallisation and the development of improved nucleating agents that can enable better anisotropic control of the reinforced polymer composites. Also, there should be focus on advancing the models to account for temperature-dependent nucleation and growth mechanisms in fibre-reinforced polymer composites. This advancement will offer better prediction methods for industry-related thermal profiles and fibre configurations.

## 4. Conclusions

A set of characterisation methods has been thoroughly utilised to obtain the TCP material properties which were largely unknown. The FTIR revealed the confirmation of the polyethylene functional group with varying amounts. The presence of the amine group can be attributed to the siliconization of glass fibre to make it hydrophobic and improve fibre-to-matrix compatibility. The inner layer was identified as HDPE based on characteristic peaks such as 2913.50 cm^−1^ and 2846.66 cm^−1^, indicating C-H stretch vibrations and the absence of significant fibre content. The reinforced layer exhibited similar PE characteristics but with additional amine groups, suggesting enhanced fibre-matrix compatibility due to potential silane treatment, aligning with LDPE-like properties but differing in density. The outer layer, while sharing some spectral similarities with the inner layer, lacked amine groups and displayed minimal fibre content, further confirming its HDPE nature and resistance to external degradation. These findings underscore the predominance of PE across the layers, with variations in functional groups and fibre treatments tailored to specific performance requirements. Using the TGA, the whole pipe thermally degrades at ~200 °C with full matrix degradation expected at 500 °C, while a double melting peak was observed from the DSC, indicating the thermal sensitivity of the HDPE matrix. The DSC also inferred that the presence of fibre facilitates transcrystallisation, which subsequently influences anisotropy. In terms of the crystallinity kinetics, the thermal stability analysis revealed that both samples experienced similar weight loss transitions, primarily occurring at ~200 °C, with full matrix degradation expected at 500 °C.

The glass fibre component remained stable up to approximately 625 °C. Based on the resin burn-off technique, the glass fibre volume fraction in the composite was estimated to be within 33–40%. DSC analysis showed that all samples underwent glass transition and exhibited melting peaks. Recrystallisation occurred in cycle 2, indicating the presence of HDPE crystals orientated on fibre surfaces. The degree of crystallinity increased with sample mass, suggesting enhanced nucleation and crystal density. The thermal behaviour of individual TCP layers was also investigated. The liner layer exhibited higher thermal stability, while the reinforced layer showed the highest degree of crystallinity due to the significant glass fibre content. Increasing fibre content raised the degradation temperature, as observed in similar studies, which infers that the presence of fibre facilitates transcrystallisation, which subsequently influences anisotropy. Also, a double melting peak was observed from the DSC, indicating the thermal sensitivity of the HDPE matrix.

Non-isothermal crystallisation kinetics were evaluated using the Avrami, Ozawa, and Mo methods to understand the variation in cooling rate (10 °C/min and 50 °C/min). Avrami and Mo methods provided insights into crystallisation rates and nucleation effects of glass fibres by enabling the identification of the crystallisation rate constant for the crystallisation rate of all the layers and confirmed that the glass fibre served as a nucleating agent that correlates to a 3D growth for homogeneous nucleation. The Ozawa method, however, showed inconsistencies in providing the crystallisation kinetics. Overall, the presence of glass fibres influenced the thermal and crystallisation behaviour of the TCP, with higher fibre content leading to increased thermal stability and crystallinity. The key finding here is that the non-isothermal crystallisation can be correlated with anisotropy. Despite the homogeneous and uniform initiation of nucleation in non-isothermal crystallisation, the resulting 3D growth displays anisotropic patterns. This anisotropic growth is not inherently due to the low crystallographic properties of glass fibre but rather the rate of supercooling during the process. Therefore, to optimise the anisotropic properties of the reinforced layer, controlling the cooling rate is vital.

This study has demonstrated that the characterisation techniques used for this work are fundamentally useful in deriving the thermo-physical behaviour information. Future research will explore the application of these results in determining the dynamic behaviour and effect of thermal expansion and shrinkage on the TCP layers. Further study worth considering should involve the precise quantification of residual thermal stress for the TCP layers in an enclosed condition, which should align with the material shrinkage dynamics. The thermal influence of the manufacturing approaches on the TCP quality during the processing should also be investigated.

## Figures and Tables

**Table 1:** Determination of glass fibre volume fraction.

Samples	Mass (mg)	Matrix Degradation (%)	Weight of Matrix (mg)	Weight of Fibre (mg)	Fibre Volume Fraction
1	29.00	65.94	19.12	9.88	0.34
2	7.60	67.42	5.12	2.48	0.33
3	8.95	60.00	5.37	3.58	0.40

**Table 2:** Degree of crystallinity for full samples.

Sample	Mass (mg)	Tm (°C)	ΔHf (J/g)	ΔHc (J/g)	Xc (%)
4	9.70	130.38	13.72	12.92	27.30
5	7.90	131.14	12.46	10.76	58.02
6	8.70	121.24	12.08	10.63	49.48

**Table 3:** Degree of crystallinity for the layers.

Sample	Mass (mg)	Tm (°C)	ΔHf (J/g)	ΔHc (J/g)	Xc (%)
Coating	9.00	120.30	17.11	16.26	29.10
Reinforced	8.90	120.17	4.31	4.19	40.95
Liner	8.10	120.10	16.94	16.56	12.96

**Table 4:** T_onset_, T_peak_, ΔH, t_1/2_, Z_c_, and n variables at the two varying cooling rates.

Sample	φ (°C/min)	T_onset_ (°C)	T_peak_ (°C)	T_onset_ − T_peak_ (°C)	Δ*H*c (J/g)	Xc	t_1/2_ (min)	*Z_c_*	n
Liner (8.10 mg)	10	118.70	115.57	3.13	16.56	12.96	4.443	2.2377	0.5518
	50	113.5	94.89	18.61	13.59	10.64	1.30	0.1776	2.0585
Reinforced (8.90 mg)	10	118.34	116.78	1.56	4.92	40.95	4.322	1.295	0.4252
	50	113.71	102.54	11.17	4.34	42.42	1.15	1.5994	0.7355
Coated (9.00 mg)	10	118.73	115.22	3.51	16.26	29.10	4.478	2.1343	0.5846
	50	116.31	96.91	19.39	14.15	25.32	1.26	0.3765	2.5287

**Table 5:** Ozawa’s parameters for all the layers.

T (°C)	Liner		Reinforced		Coating	
	m	ln Z(t)	m	ln Z(t)	m	ln Z(t)
60	0.2319	0.473	1.1957	0.6255	0.1036	0.5315
80	1.4327	0.8336	1.6028	0.6923	4.2816	1.4924
100	0.5797	0.0609	0.6729	0.1959	0.0203	0.1638
120	1.1631	0.0197	1.8335	0.6545	0.8599	0.0569
140	0.2409	0.379	2.2271	0.8032	0.0415	0.389

**Table 6:** Non-isothermal crystallisation kinetic parameters from the Mo method.

Xt (%)	Liner		Reinforced		Coated	
	F(T)	α	F(T)	α	F(T)	α
20	1.9811	0.9113	0.1584	0.6385	3.2957	1.243
40	3.4197	1.4117	2.3548	0.9651	3.3576	1.2672
60	3.8758	3.1817	2.9623	1.0122	3.709	1.8801
80	3.9014	3.9148	3.9918	1.0281	5.0128	1.6178

## Data Availability

The original contributions presented in this study are included in the article. Further inquiries can be directed to the corresponding author.

## References

[1] Soleimani M., Gholami R., Alijani A., Ansari R. (2023). An analytical solution for dynamics of cyclic thermomechanically loaded multi-layered filament-wound composite pipes in hygrothermal environment. Thin-Walled Struct..

[2] Sulu I.Y., Temiz S. (2020). Mechanical characterization of composite pipe systems joined using different radii pipes subject to internal pressure. Mech. Based Des. Struct. Mach..

[3] Bahaman U.S.F., Mustaffa Z., Seghier M.E.A.B., Badri T.M. (2024). Evaluating the reliability and integrity of composite pipelines in the oil and gas sector: A scientometric and systematic analysis. Ocean Eng..

[4] Mahdavi H., Rahimi G.H., Farrokhabadi A. (2021). Fatigue performance analysis of GRE composite pipes by conducting tension-tension tests on the rings cut from the pipe. J. Test. Eval..

[5] Okolie O., Latto J., Faisal N., Jamieson H., Mukherji A., Njuguna J. (2023). Manufacturing defects in thermoplastic composite pipes and their effect on the in-situ performance of thermoplastic composite pipes in oil and gas applications. Appl. Compos. Mater..

[6] Okolie O., Latto J., Faisal N., Jamieson H., Mukherji A., Njuguna J. (2023). Advances in structural analysis and process monitoring of thermoplastic composite pipes. Heliyon.

[7] Saghir F., Gohari S., Mozafari F.A.R.Z.İ.N., Moslemi N., Burvill C., Smith A., Lucas S. (2021). Mechanical characterization of particulated FRP composite pipes: A comprehensive experimental study. Polym. Test..

[8] Phani K.V.S., Nanda B.K., Mishra S.B., Nayak S.K. (2023). Structural analysis of experimentally fabricated glass fiber reinforced (GFRP) composites. Mater. Today Proc..

[9] Akın Y., Kara M. (2024). Mechanical strength and low-velocity impact behavior of glass fiber reinforced filament wound pipes with different number of layers after hydrothermal aging. J. Compos. Mater..

[10] Alkan U., Özcanlı Y., Alekberov V. (2013). Effect of temperature and time on mechanical and electrical properties of HDPE/glass fibre composites. Fibres Polym..

[11] Behboudi A., Jafarzadeh Y., Yegani R., Akbari A. (2017). Preparation and characterization of polyethylene/glass fibre composite membrane prepared via thermally induced phase separation method. Polyolefins J..

[12] Siddique S.A. (2020). Processing, Structure and Thermo-Mechanical Properties of Reclaimed Nanoclay, and its Application in Polyamide 6 and Low-Density Polyethylene Nanocomposites. Ph.D. Thesis.

[13] Maheswari C.U., Reddy K.O., Muzenda E., Shukla M., Rajulu A.V. (2013). A comparative study of modified and unmodified high-density polyethylene/borassus fibre composites. Int. J. Polym. Anal. Charact..

[14] Hu P., Zhao P.P., Zhang G.W., Wang X.H. (2016). Thermal properties of 60Co irradiated crosslinked high density polyethylene. Sol. Energy Mater. Sol. Cells.

[15] Awad A.H., Abd El-Wahab A.A., El-Gamsy R., Abdel-latif M.H. (2019). A study of some thermal and mechanical properties of HDPE blend with marble and granite dust. Ain Shams Eng. J..

[16] Batista N.L., Olivier P., Bernhart G., Rezende M.C., Botelho E.C. (2016). Correlation between degree of crystallinity, morphology and mechanical properties of PPS/carbon fibre laminates. Mater. Res..

[17] Wang Y., Cheng L., Cui X., Guo W. (2019). Crystallization behavior and properties of glass fibre reinforced polypropylene composites. Polymers.

[18] Liu B., Wu W. (2019). Nonisothermal crystallization kinetics of poly (butylene terephthalate)/epoxidized ethylene propylene diene rubber/glass fiber composites. Polym. Eng. Sci..

[19] Tong X., Wang Z., Zhang M.L., Wang X.J., Zhang G., Long S.R., Yang J. (2020). Synthesis, characterization and non-isothermal crystallization kinetics of a new family of poly (ether-block-amide) s based on nylon 10T/10I. Polymers.

[20] Mahmud N.F.F. (2014). A Study on Mechanical and Thermal Properties of High Density Polyethylene (HDPE) Glass Fibre Composites.

[21] Miao W., Zhu H., Duan T., Chen H., Wu F., Jiang L., Wang Z. (2018). High-density polyethylene crystals with double melting peaks induced by ultra-high-molecular-weight polyethylene fibre. R. Soc. Open Sci..

[22] Wang P., Lin Q., Wang Y., Liu C., Shen C. (2021). Comparative study of the crystallization behavior and morphologies of carbon and glass fiber reinforced poly (ether ether ketone) composites. Polymers and Polymer Composites..

[23] Jeziorny A. (1978). Parameters characterizing the kinetics of the non-isothermal crystallization of poly (ethylene terephthalate) determined by DSC. Polymer.

[24] Xu W., Liang G., Wang W., Tang S., He P., Pan W.P. (2003). Poly (propylene)–poly (propylene)-grafted maleic anhydride–organic montmorillonite (PP–PP-g-MAH–Org-MMT) nanocomposites. II. Nonisothermal crystallization kinetics. J. Appl. Polym. Sci..

[25] Alms J., Hopmann C., Wang J., Hohlweck T. (2020). Non-isothermal crystallisation kinetics of polypropylene at high cooling rates and comparison to the continuous two-domain pvT model. Polymers.

[26] Tadokoro D., Konishi T., Fukao K., Miyamoto Y. (2023). Lamellar crystallization of poly (trimethylene terephthalate). Polym. J..

[27] Boukettaya S., Al Seddique W., Alawar A., Daly H.B., Hammami A. (2016). Cooling rate effects on the crystallization kinetics of polypropylene/date palm fibre composite materials. Sci. Eng. Compos. Mater..

[28] Huang J.W. (2008). Isothermal crystallization of high density polyethylene and nanoscale calcium carbonate composites. J. Appl. Polym. Sci..

[29] Li D., Luo C., Zhou J., Dong L., Chen Y., Liu G., Qiao S. (2023). The role of the interface of PLA with thermoplastic starch in the nonisothermal crystallization behavior of PLA in PLA/Thermoplastic Starch/SiO_2_ Composites. Polymers.

[30] Ozawa T. (1971). Kinetics of non-isothermal crystallization. Polymer.

[31] Liu M., Zhao Q., Wang Y., Zhang C., Mo Z., Cao S. (2003). Melting behaviours, isothermal and non-isothermal crystallization kinetics of nylon 1212. Polymer.

[32] Yuan Q., Awate S., Misra R.D.K. (2006). Nonisothermal crystallization behaviour of polypropylene–clay nanocomposites. Eur. Polym. J..

